# Preparedness of health care professionals in preventing maternal mortality at a public health facility in Ghana: a qualitative study

**DOI:** 10.1186/s12913-016-1527-y

**Published:** 2016-07-12

**Authors:** Hubert Amu, Samuel H. Nyarko

**Affiliations:** Department of Population and Health, University of Cape Coast, Cape Coast, Ghana; Department of Population and Behavioural Sciences, School of Public Health, University of Health and Allied Sciences, Hohoe, Ghana

**Keywords:** Preparedness, Health professionals, Maternal mortality, Ghana

## Abstract

**Background:**

Preparedness of health care professionals for emergency situations is quite indispensable in quality health care; yet, information barely exists on the preparedness of health care professionals for emergency cases in health facilities in Ghana. This study sought to assess the preparedness of health professionals in preventing maternal mortality cases at a public health facility in Ghana.

**Methods:**

This is a qualitative study that used purposive and convenient sampling techniques to recruit 12 health care professionals for in-depth interviews. The interviews were tape-recorded, transcribed and the results presented in themes verbatim.

**Results:**

The results show that health care professionals at the hospital were ill-equipped to effectively handle maternal mortality cases in the municipality. As such, inadequate staff size and dearth of essential logistics militate against the preparedness of health care professionals in curbing maternal mortality.

**Conclusions:**

More health care professionals and essential logistics should, therefore, be provided by the Ghana Health Service to augment the existing ones.

**Electronic supplementary material:**

The online version of this article (doi:10.1186/s12913-016-1527-y) contains supplementary material, which is available to authorized users.

## Background

In spite of the advancement in public health and medical care, about 287,000 women still die globally each year from causes linked to pregnancy and childbirth [1]. As such, about 785 women die daily and more than 32 die every hour. While 99% of these deaths occur in developing countries worldwide, about 85% occur in sub-Saharan Africa and South Asia alone [1]. Hence, maternal mortality is considered the leading cause of death among females aged 15–49 years old globally [2].

Ghana's maternal mortality ratio remains high even though efforts have been made over the past two decades by the country and partner institutions including the World Health Organisation (WHO) to reduce it [2]. According to the WHO, UNICEF, UNFPA, The World Bank and the United Nations Population Division [3], the country’s maternal mortality is high (319 per 100,000 live births). Hence, it was obvious that the country was unable to achieve the MDG-5 target by 2015 [3]. This, therefore, gave rise to 17 newly created sustainable development goals, a target of goal three (to ensure healthy lives and promote well-being for all at all ages) which requires Ghana to reduce her maternal mortality ratio to less than 70 per 100,000 live births by 2030 [4].

In the district where the study health facility operates, maternal mortality was found to be quite high. Statistics indicate that in 2013, the district recorded one of the highest maternal mortality cases in the country [5]. This may be attributed to the inadequacy of midwives and doctors who are supposed to handle maternal cases as well as the lack of essential logistics for handling such cases. A considerable number of studies have been conducted on maternal mortality in Ghana. Aside the Ghana Demographic and Health Surveys (1988, 1993, 2003 and 2008) and the UNICEF Multiple Indicator Cluster Surveys (MICS) among others which provided general statistics on maternal mortality, Asamoah et al. [2] also examined the distribution of causes of maternal mortality among different socio-demographic groups in Ghana. However, it has been observed that there is limited information on the preparedness of health care professionals in preventing maternal mortality cases in Ghana, including the study district. Consequently, this study sought to assess the preparedness of health care professionals in preventing maternal mortality cases at a public health facility, Ghana. Specifically, the study sought to examine the availability of human resources and essential logistics in preventing potential maternal mortality cases as well as explore factors contributing to high maternal mortality cases at the health facility.

## Methods

The study was purely qualitative and a case study research design was adopted. Sorrell and Redmond [6] argue that case study is related to the in-depth analysis of a single or a small number of units of a scenario or issue. Consequently, it is useful in conducting an in-depth assessment of the preparedness of health care professionals in handling maternal mortality cases at a public health facility in Ghana.

Purposive and convenient sampling techniques were used in sampling respondents. The purposive sampling was meant to ensure that only those relevant to the study (health professionals) were contacted, since they are considered more knowledgeable and abreast of the subject of interest [7]. In selecting participants to be included in the study, convenient sampling technique was used to recruit 12 health care professionals for in-depth interviews. This comprised four medical doctors including the medical superintendent (head) of the hospital, three midwives, three nurses, one pharmacist and one laboratory technician.

An in-depth interview guide with 22 questions was used to collect data from the participants (Additional file 1). The instrument was divided into three sections; A-C. Section A was based on the socio-demographic characteristics of the respondents (sex, age, level of education, religion, marital status and duration of working at the facility). Section B looked at the availability of health professionals and essential logistics in handling maternal mortality cases. Issues addressed in this section comprised the number of maternal cases reported to the hospital in a week, adequacy in a number of health care professionals and availability essential drugs and resuscitation materials. The section also addressed issues on the availability of equipment and whether maternal deaths at the hospital could be attributed to the inadequacy and unavailability. The third section also focused on factors contributing to maternal mortality at the facility. Issues discussed in this section included receiving of antenatal education and adherence to education received by pregnant women as well as the attitude of health care providers towards pregnant women.

Data were collected by two experienced research assistants. For the purpose of this research, the assistants were trained for two days (four hours for each day) to adequately acquaint them with the instrument. Data were collected for two weeks with each interviewee being interviewed only once. The interviews lasted generally between 30 to 40 min. All interviews were tape-recorded, transcribed and edited, and the texts were printed. Inductive content analysis was used in analysing the data. After reading through all the 12 transcripts a number of times, themes were identified. This led to the generation of codes based on the meanings of the emerged themes. This led to the creation of a data-driven codebook which had 9 major codes which were created by one person. Even though the codes were created by only one of the researchers, a peer debriefing process was involved in the coding development process. This helped to reduce the possibility of researcher bias in the development of the codes. The codes were then revised in the context of the data. The results were presented in quotes to substantiate issues discussed.

## Results

### Socio-demographic characteristics of respondents

Table 1 presents socio-demographic characteristics of the respondents. While females constituted 66.7 %, males formed 33.3 %. While respondents who were in their 20s and 40s constituted 16.7 % respectively, those who in their 30s and 50s also formed 33.3 % each. All respondents had tertiary levels of education (100 %), were generally Christians (75 %) and married (66.7 %). One-fourth of the health professionals had worked at the hospital for less than a year prior to the data collection while close to one-tenth (8.3 %) had worked for more than 10 years. Those who worked at the hospital for 1–5 years and 6–10 years also constituted 41.7 % and 25 % respectively (Table 1).

**Table 1:** Socio-demographic characteristics of respondents

Characteristic	Frequency	Percent (%)
Sex		
Male	4	33.3
Female	8	66.7
Age		
20–29	2	16.7
30–39	4	33.3
40–49	2	16.7
50–59	4	33.3
Level of education		
Tertiary	12	100.0
Religion		
Christian	9	75.0
Muslim	3	25.0
Marital status		
Never married	2	16.7
Married	8	66.7
Divorced	1	8.3
Widowed	1	8.3
Duration of working at the hospital		
<1 year	3	25.0
1–5 years	5	41.7
6–10 years	3	25.0
>10 years	1	8.3
Total	12	100.0

Source: Field work, 2014

### Availability of human resources and essential logistics for preventing maternal mortality cases

The number of maternal cases reported to the hospital were explored. Cases reported generally comprised haemorrhage, maternal sepsis, gestational diabetes and preeclampsia. While some respondents gave estimates, others were unsure. Respondent 2 (50 years), for instance, reported: *about 200 cases come a week.* Respondent 4 (33 years), however, reported: *since maternal cases form part of the general services we provide, we may not be able to differentiate between maternal and other cases.*

When asked whether there were enough professionals to handle maternal cases that they receive at the hospital, all the respondents indicated that there were not enough at their various units. The following quote summarises their responses:

*“Yes! There is an inadequacy in terms of the size of staff we have here. This is because we have a room for handling minor cases and another for major cases. Sometimes, we have three nurses on duty in the morning shift, and one person can be assigned to the minor operating room while the other two people can be assigned to the major operating room. So when more hands are needed and somebody is busy, they would have to leave there and come. But on most occasions, there is still not enough personnel. We have to call on the afternoon staff from their homes. And sometimes, we do not even get the extra hand that is needed. So yes, in this context, there is the inadequacy of staff”* (Respondent 6, 32 years).

Respondents were further asked whether there had ever been a situation where health professionals available were not enough to handle the number of maternal cases brought to the facility within a particular time. They all responded in the affirmative. One respondent, for instance, had this to say:

*“Yes! It (inadequacy of health professionals) is a contributing factor. Because, in handling a lot of cases, like here in the theatre, you need team work. So if you do not have a lot of staff around, then there is a problem. Sometimes too, it (inadequacy of health professionals) leads to inadequate monitoring. You see? We usually have the situation where instead of one person monitoring a mother in labour closely every 15 min, because of too much work, they are not able to do that. Complications, therefore, arise out of that leading to maternal deaths”* (Respondent 5, 54 years).

With regard to availability of essential logistics, respondents generally indicated that they had enough drugs and anaesthetic agents to handle maternal cases brought to the facility. Respondent 3 (52 years), for instance, reported: *Yes, for drugs, we have enough. Normally, we collect some drugs like magnesium sulphate, oxytocin and intravenous fluid and put them in our ward stock.* Respondent 4 also reported:

*“Yes, we have enough (anesthetic agents); we have never run short of them. Currently, we are also using ketamine and devakane (hevimakane) as our main anesthetic agents. Other supplementary drugs are atropine, pethidine and so on. We have never run short of those anesthetic agents. We always make sure that before they get finished, we had a replacement. And the intravenous fluids too, we have always ensured that they are available”* (Respondent 4, 33 years).

Respondents also indicated that they had enough oxygen to handle maternal emergency cases. Respondent 5 (54 years) reported: *For oxygen, it is not a problem now because we have concentrators which we can use when there is power*. Respondent 3, however, noted: *we have only one oxygen cylinder, so if two people need oxygen you can imagine what will happen here. Ergometrine and Sulphadoxine Paramitamine (SP) are also not available*. Respondent 1 in this regard reported: *No! It is only ergot (Ergometrine) we do not have. For about a year now, it has been out of the system.* Respondent 2 (50 years) also reported: *I can say that for some time now, we have been short of Sulphadoxine Paramitamine.*

Availability of blood was also a major challenge indicated by all the respondents. Respondents explained that shortage of blood had been a constraint to the successful delivery service during emergency cases. For instance, one respondent reported:

*“....but blood is not always available. When you go to the blood bank, they will not be able to give it to you. The relatives’ blood are usually not compatible or sometimes do not have enough to donate. So in that case, there is normally a problem”* (Respondent 7, 29 years).

When asked whether maternal mortality at the hospital could be attributed to drugs and resuscitation materials, respondents generally indicated that maternal deaths could not be attributed to oxygen and drugs, but to others such as blood, ergometrine and SP, since they were the ones in shortage. In view of this, one of the respondents, however, disagreed with the others and blamed maternal deaths on the efficiency of the nursing staff. He reported:

*“It is not always about unavailability but the staff preparing for emergency cases always, you understand. When nurses go off duty, as part of their shift handing over, they have to hand over every equipment such as the suction machine, the oxygen and the other monitors. Sometimes, somebody may be vomiting and it is then that they are going to look for a suction machine. They may not know where it is at that moment. Whether it was used for somebody somewhere or maybe they dismantled it and had not yet set it up, no one knows. In this case, it is not only about the availability but the emergency preparedness that is accorded to maternal emergency cases by the staff”* (Respondent 5, 54 years).

Equipment such as operation sets was reported by respondents to be always available. One respondent, for instance, reported:

“*Yes, we always make sure that we have our instruments. They are always intact. Even when there is none, in all the various surgeries that we had, for instance, the hernia myomectomy, we have sets which we use. If the caesarean section (CS) set is exhausted, we normally break other sets to get instruments for the CS”* (Respondent 7, 29 years).

When asked whether logistics, on the whole, were adequate for handling maternal cases, respondents generally alluded to the issues they raised initially with regards to availability of drugs, blood, and other resuscitation materials and noted that while some were adequate others were inadequate. They further explained that the inadequate supply of some of these logistics together with staff availability and preparedness result in most of the maternal deaths at the facility. In view of this, one respondent, however, blamed the staff for not properly handling such logistics to save lives:

*“The few equipment and other logistics we have are available, if the staff can put them in right use, it will save lives. But, issues concerning the availability of staff, preparedness and human errors which occasionally come in, are the some of the challenges we face”* (Respondent 5, 54 years).

### Factors contributing to high maternal mortality at the facility

To ascertain factors contributing to maternal mortality at the facility, respondents were asked several questions including health education and waiting time of women before they were attended to. Respondents were, for instance, asked if women were usually given education on proper nutrition, treatment of minor ailments and the need for a regular antenatal visit. All the respondents reported that the women were usually educated. A respondent then indicated:

“*Yes! Education is given individually according to the patient’s needs. We educate each mother after examination and palpation. So, whatever we think they need to know, we teach them”* (Respondent 2, 50 years).

The respondents further explained that even though education was usually given, the women did not usually conform to it. When asked whether women normally conformed to the education given them, respondent 1 (24 years), for instance, reported:

*No! Sometimes, they do not. I do not know what is wrong with most of them. They undermine the education given them about the emergency of the situation. They do not see it to be realistic until it happens to them.*

A respondent, however, attributed non-conformity to religious beliefs of the people. She had this to say: *The people here believe in traditional worship and when something is wrong with them, they attribute it to the gods. So when you teach them, some take it and others do not* (Respondent 3, 52 years).

The respondents indicated that waiting time was long and they attributed the situation to their inadequacy in terms of staff numbers. The following was the report made by one of the respondents:

*“Ideally, if you come to the antenatal clinic (ANC), after one hour you should leave, but the two midwives there have retired and are on contract. So the number of mothers who come there are too many to handle and that is how come the mothers waste time”* (Respondent 12, 49 years).

The respondents reported that owing to shortage of blood at the facility, they normally asked relatives to donate blood prior to blood transfusion for the mothers/pregnant women. Some husbands of the women according to respondents, usually brought their wives to the hospital and even stayed with them during labour and delivery. Sometimes, however, due to the number of women in the labour ward, the men were not allowed to be with their wives. One respondent, for instance, explained:

*“For that one, yes and no. Yes because we have only three labour wards. The way the places are, when there are other women there, the husbands (of the women) cannot come, but when she is alone in the labour ward, we do allow them”* (Respondent 1, 24 years).

Respondents indicated that maternal mortality was usually as a result of late reporting by pregnant women. The health care professionals indicated that pregnant women normally waited until they had complications before they report to the hospital. The following statement summarises their responses:

“*Yes! As for that yes. The women here are stubborn. They sit at home and when they are unable to deliver, they then come here in a deteriorated condition. That is the problem we face here. They will do their own things at home and when the condition is worse then they come and die here”* (Respondent 3, 52 years).

According to respondents, the hospital pharmacy provided a 24-h service. However, the laboratory did not and this posed problems sometimes. One respondent explained: *Oh! We do not open the pharmacy 24 hours… but we have somebody on standby. In the case of any emergency, the person is called to attend to whatever situation is at hand.* (Respondent 8, 32 years).

Respondents have reported other institutional challenges contributing to maternal deaths at the facility to include lack of staff motivation, the inadequacy of vehicles for hospital activities especially during emergencies, and lack of emergency preparedness. Consequently, when maternal emergency cases were brought, it became very difficult for them to handle. With regard to emergency preparedness, one respondent had this to say:

*“The equipment may be there, but if we are not always prepared for emergency cases, it becomes a problem. Whenever emergency cases come, we will now be running to search for equipment and then becomes difficult to find. Later on, you will realize that it was lying somewhere. I am very sure that if I go now and ask any of the staff whether they have magnesium sulphate, they may say they do not know. So, it is the way we are prepared mentally to respond to emergency cases”* (Respondent 4, 33 years).

Some of the respondents also conceded that negative attitude of staff towards pregnant women deterred them from regular attendance to antenatal clinic and hospital delivery which subsequently resulted in maternal deaths. One of the respondents had this to say:

*“Sometimes, when they come to the facility, the way some of us doctors and midwives talk to them, we abuse them. Especially teenage pregnancy, when they come the way we insult them; like, ‘you small girl, why?’ So next time, when something is happening, she would not want to come back”* (Respondent 5, 54 years).

## Discussion

Findings of the study show that while health care professionals were not sure of the average number of maternal cases reported at the hospital in a week, reported figures ranged from 100 to 200 cases. In spite of the high number of maternal mortality cases reported at the facility, health care professionals in this study were inadequate in handling such cases. Hence, some of them resulted in maternal mortality. Hill et al. [8] argued in this regard that in many countries, particularly the developing ones (including Ghana), health care providers are usually in limited supply in addition to essential logistics needed to save lives.

Essential logistics such oxygen and drugs including oxytocin*,* intravenous fluid, magnesium sulphate were reported to be always available to handle maternal cases brought to the hospital. The hospital, however, had only one oxygen cylinder. So, if two people needed oxygen at the same time, it became problematic. The major challenge with regards to essential logistics as revealed by the study has to do with non-availability of blood, Ergometrine and Sulphadoxine Paramitamine. Respondents, therefore, attributed maternal mortality at the Hospital to non-availability of such essential logistics. These findings confirm Jensen and Rappaport’s [9] argument that not only are health care professionals inadequate in terms of their numbers to handle maternal mortality, but non-availability of essential logistics including blood also serve as major impediments that hamper the preparedness of health care professionals to handle maternal mortality.

In terms of factors contributing to high maternal mortality at the Hospital, the study revealed that even though the women were regularly given education on all issues of proper maternal health including handling minor complications and proper nutrition, the challenge, however, is the fact that majority of the women did not conform to the education given them, which results in maternal deaths. It also came out that long waiting time at the hospital leads to delays in accessing maternal care, which the health professionals admitted, results in maternal deaths. Another major factor which results in maternal mortality at the hospital is late reporting of pregnant women to the health facility for antenatal care. As a result, some of them die upon reaching the hospital. Confirmed by this finding is a study conducted by Ndidi and Oseremen [10] which indicated that while some women do not attend antenatal care at all, others start reporting very late into their pregnancies. The results as noted by Jensen and Rappaport [9] are that these women usually develop complications and are rushed to the nearest health care facilities with some of the complications resulting in the death of such women.

Emergency preparedness was a major organisational factor indicated as being responsible for maternal mortality at the study facility. This is consistent with Amollo [11] who stated that unpreparedness of health care professionals usually during emergency situations, results in maternal deaths, particularly in developing countries. Aside inadequate emergency preparedness, however, there was a lack of staff motivation and inadequacy of vehicles for hospital activities, particularly during emergencies. The lack of logistics realised by the present study confirms Frimpong Manso Annan and Anane’s [12] argument that Ghana is faced with a plethora of health challenges including lack of logistics and health service providers for effective health care delivery.

The fact that the study was conducted in only one health facility and used a relatively small sample size greatly limited its generalizability. This does, however, not limit the validity and reliability of the study in any way.

## Conclusions

The study revealed that health care professionals at the study facility were not well-equipped to handle maternal emergency cases brought to the facility which in turn resulted in a number of maternal mortality cases. Inadequacy in terms of health care professionals and essential logistics constrained the level of preparedness of health care professionals in handling potential maternal mortality cases in the health facility. Non-adherence to health education given by health care professionals and delay in reporting complications on the part of patients also posed a major challenge to reducing maternal mortality cases in the facility. It is therefore imperative for Ghana Health Service in collaboration with non-governmental organisations to add to the staff of the hospital and provide adequate essential logistics such as blood, oxygen cylinders, ergometrine and sulphadoxine paramitamine to the health facility, intensify education through community outreach and adequately motivate the health care professionals in order to boost their preparedness in preventing maternal mortality cases in the district.

## Abbreviations

CS, Caesarean Section; MDG, Millennium Development Goal; MICS, Multiple Indicator Cluster Surveys; SP, Sulphadoxine Paramitamine; UNICEF, United Nations Children's Fund; WHO, World Health Organisation

## Additional file

Additional file 1: Interview guide for health workers. (DOC 30 kb)
