# Supplementary material for: Preparedness of health care professionals in preventing maternal mortality at a public health facility in Ghana: a qualitative study
Source: BMC Health Serv Res. 2016 Jul 12;16:252. doi: 10.1186/s12913-016-1527-y (PMC4942930; doi:10.1186/s12913-016-1527-y)
Supplement: Additional file 1: — Interview guide for health workers. (DOC 30 kb) [file 12913_2016_1527_MOESM1_ESM.doc]

**INTERVIEW GUIDE FOR HEALTH WORKERS**

Good evening and welcome to the discussion. Thank you for taking the time to join me to talk about **Preparedness of health care professionals in preventing maternal mortality at your facility.** You have been included in this study because you are a health worker and considered vital in understanding issues relating to maternal mortality and health workers’ preparedness to handle it. I would, therefore, be happy if you would provide frank answers to the questionnaire items. You are fully assured of anonymity and confidentiality of all information provided.

**Section A: Socio-demographic data**

1. Sex:______________
2. Age (in completed years):_____________
3. Level of education________
4. Religion:_____________
5. Marital status:_________
6. How many years have you worked in this hospital? ______________

**Section B: availability of health professionals and essential logistics in handling maternal mortality cases**

1. On average, how many maternal cases are brought to the hospital in a week?
2. Are you midwives/nurses/doctors/pharmacists enough to handle the maternal cases reported at the hospital every week? (Probe for the average midwives/doctors-patient ratio)
3. Has there been a situation where health professionals available were not enough to handle the number of maternal cases brought to the hospital within a particular time? (probe for day, month and/or year).
4. Can any maternal death at this hospital be attributed to inadequacy of healthcare professionals to handle such cases?
5. Does the hospital have essential drugs for use particularly in emergency maternal situations (Probe for magnesium sulphate, oxytocin and intravenous infusion)?
6. Are resuscitation materials (oxygen, magnesium sulphate, ergometrine and blood) always available and enough to handle maternal cases especially in emergencies?
7. Would you attribute any maternal mortality at the hospital to non-availability of drugs, or resuscitation materials to handle maternal cases at the hospital (Probe for particular instances of maternal mortality resulting from those situations).
8. Are equipment including operation set always readily available for surgery whenever there is the need to use them?
9. How would you generally describe the availability of essential logistics for the reduction of maternal mortality at the hospital? (probe for very adequate, adequate, not adequate and not at all adequate).

**Section C: Factors contributing to maternal mortality**

1. Are pregnant women usually given education on proper nutrition, treatment of minor ailments and the need for regular antenatal visit? (Probe for minor ailments including hypertension, diabetes, haemorrhage and malaria)
2. In your assessment, would you say that pregnant women usually conform to education given them at the hospital?
3. Do relatives of the pregnant women have to donate blood prior to the woman’s delivery?
4. Do you allow husbands to be with their wives during labour and delivery?
5. Can maternal mortality at this hospital be attributed to late reporting for antenatal care by mothers?
6. Does the pharmacy and laboratory operate for 24 hours a day? (Probe for regular shifts run by the pharmacists and technicians).
7. Which other factors can maternal mortality at this hospital be attributed to?

Thank you for participating in this study.
